# The role of prophylactic iron utilization and nutrition to prevent iron deficiency in infancy: Prospective cohort study

**DOI:** 10.1097/MD.0000000000042947

**Published:** 2025-06-13

**Authors:** Ayşe Şimşek, Vesile Meltem Energin

**Affiliations:** a Department of General Pediatrics, Konya City Hospital Pediatric Hematology and Oncology Clinic, Konya, Turkey; b Department of General Pediatrics, Necmettin Erbakan University Meram Medical Scholl Pediatrics Clinic, Konya, Turkey.

**Keywords:** breastfeeding, iron deficiency, iron deficiency anemia, nutrition, prophylaxis

## Abstract

Incorporating iron-rich foods into the diet, adequate breast milk intake, and prophylaxis with iron preparations can prevent iron deficiency anemia (IDA) and iron deficiency (ID) in infancy. This study aimed to elucidate the effects of prophylactic iron preparations, adequate breast milk, and iron-rich supplementary food intake on preventing IDA and ID in infancy. This prospective cohort study included 204 children aged 6 to 24 months who were admitted to the general pediatric outpatient clinic. The patients were divided into groups with and without ID and those with and without IDA. The rates of correct prophylaxis use, adequate breast milk intake, and supplementary food intake were compared. In our study, the ID and IDA rates were 53.9% (n = 110) and 34.8% (n = 71). The rate of recommendation for iron prophylaxis in the 204 patients in our study was 95.1% (n = 194). In our study, the rate of prophylactic use among all patients was 66.7% (n = 136). However, the proportion of patients who correctly used prophylaxis in the entire group was 13.7% (n = 28). The most critical risk factor for developing ID and IDA was lack of prophylaxis (*P* = .001 and odd ratio: 8.115 for ID and odd ratio: 13.364 for IDA). Prophylaxis emerged as the most critical risk factor for the development of ID and IDA in children aged 6 to 24 months. However, the consumption of more iron-rich foods and breastfeeding for the 1st 6 months is a reliable protective measure against ID and IDA, providing reassurance and confidence in the prevention of these conditions.

## 1. Introduction

Iron plays an essential role in oxidation and reduction reactions, which have a very important place in the continuation of life. Iron is crucial in these reactions because it can easily gain and lose electrons during the redox reactions. Iron is mostly found in heme molecules of organisms. Iron is structurally and functionally important for many enzymes and proteins other than heme.^[1]^ Iron deficiency (ID) is the most common nutritional deficiency among children worldwide. ID is more common in socioeconomically developing countries. However, this remains an important issue in developed countries. A plasma ferritin level below 12 mg/L is defined as ID and is often used synonymously with iron deficiency anemia (IDA). However, ID develops before anemia occurs. Weakness, fatigue, quick tiring, insomnia, and regression in neurocognitive functions may occur in ID that precedes the signs of anemia.^[2,3]^

In infants with ID, psychomotor development is adversely affected, cognitive function development decreases or stops, and immune system function regresses. Although iron treatment can reverse these adverse effects, some are permanent. Therefore, the prevention of ID is more important than treatment. Additionally, preventing ID and IDA is easier and cheaper than treating them.^[4–6]^ In our country, the Ministry of Health established a program and health policy recommending iron prophylaxis to children aged 4 to 12 months. However, data on the outcomes of this prophylaxis program are lacking.

Dietary iron-rich foods, adequate breast milk intake, and prophylaxis with iron supplements may help prevent ID and IDA. However, data on prophylactic iron supplements are controversial.^[7]^

Our primary aim in this study was to determine the effects of prophylactic iron supplements, adequate breast milk, and iron-rich supplements in preventing IDA and ID in infancy. Our secondary aim was to compare iron prophylaxis with an iron-rich diet and breast milk.

## 2. Materials and methods

### 2.1. Study design

**Figure 1.**
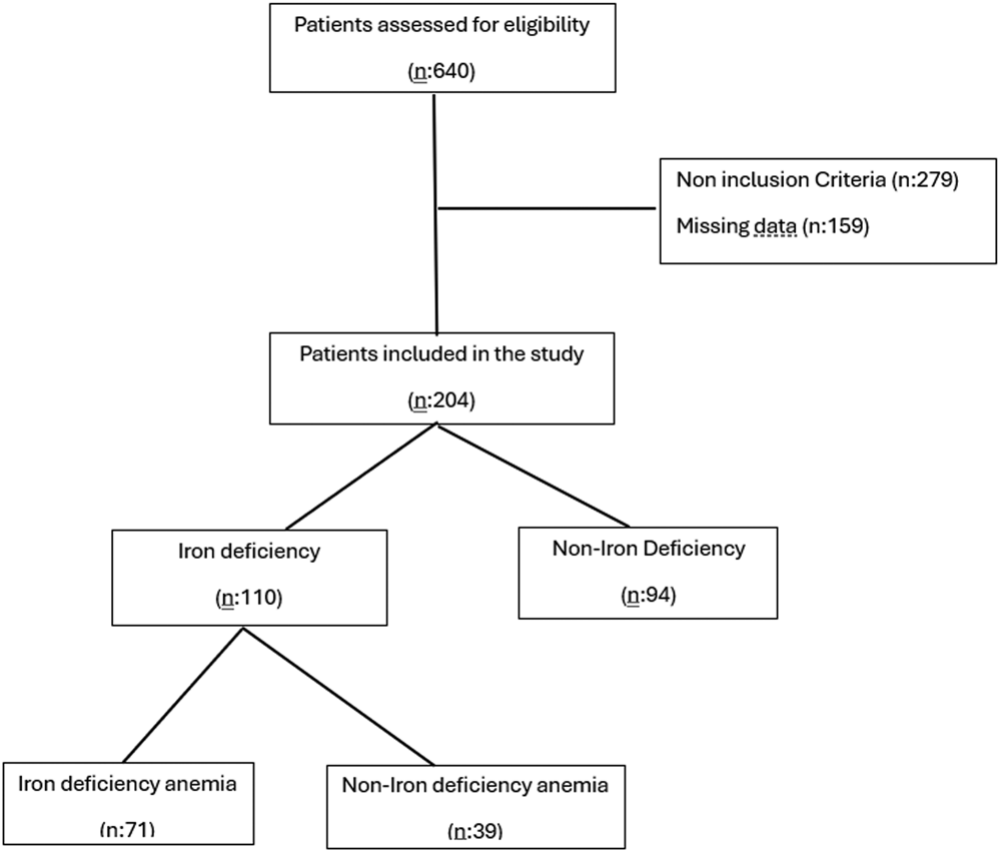
Flow diagram of participant inclusion and exclusion. This figure illustrates the process of participant selection for the study, including initial screening, application of inclusion and exclusion criteria, and determination of the final analytical sample. The number of individuals assessed for eligibility, excluded (along with reasons such as missing data or ineligibility), and ultimately included in the final analysis are presented in sequence.

A total of 640 patients were included in the study. A total of 436 patients who did not meet the criteria (n: 277) and did not have complete follow-up data (n: 159) were excluded from the study. This prospective cohort study (observational) included 204 children aged 6 to 24 months who were admitted to a general pediatric outpatient clinic. Patients were not randomized. Patient selection is shown in Figure 1 (flow chart). Patients included in the study were divided into groups with and without ID and those with and without IDA. The rates of correct prophylaxis use, adequate breast milk intake, and supplementary food intake were statistically compared between the groups. In addition, logistic regression analysis revealed the relationship between not using proper prophylaxis, not getting enough breast milk and supplementary food, and ID and IDA. Short stature and low body weight rates were compared between the groups with and without IDA.

A questionnaire was administered to determine the use of iron prophylaxis, breastfeeding, and nutritional status. In addition, serum ferritin levels, transferrin saturation, hemoglobin levels, red cell distribution width (RDW), and mean corpuscular volume (MCV) values of the patients included in the study were recorded. The same researcher completed all questionnaires to achieve a unified response. The height and weight of all participants were measured, and the Centers for Disease Control and Prevention curves and height and weight percentiles were determined. First, the ID/IDA ratio was determined in the patient group.

### 2.2. Inclusion criteria

Patients aged between 6 and 24 months who were admitted to Necmettin Erbakan University Medical Scholl pediatric outpatient clinic were enrolled in this study.

### 2.3. Exclusion criteria

Patients with chronic diseases, blood transfusion history, thalassemia carriers, clinical diagnosis of infection, or elevated C-reactive protein levels at admission were not included. In addition, patients who received ID or IDA treatment, those who were born prematurely, and those with low birth weight were excluded.

### 2.4. Evaluation

Patients with ferritin levels below 12 mg/L were considered to have ID, and those with ferritin levels below 12 mg/L and hemoglobin values below 11 g/dL were considered to have IDA. MCV value of <70 µm^3^ in children aged 6 to 24 months is considered microcytosis.^[8]^ In IDA, erythrocyte sizes are different, and this is called anisocytosis. An RDW value >16 reflects anisocytosis.^[9]^ Transferrin saturation was calculated by dividing serum iron by the serum iron-binding capacity and multiplying by 100. A transferrin saturation of <16% favor IDA.^[10]^ While evaluating the questionnaire, we determined whether prophylaxis was recommended to the patients and whether the recommended dose was used. The use of 10 mg/day iron drops from the 4th month was accepted as the correct prophylaxis recommendation. Exclusive breastfeeding for at least 6 months after delivery was considered adequate breast milk intake. In our study, those who received breast milk for less than 6 months were considered to have insufficient breast milk.

### 2.5. Ethics approval

All procedures followed were in accordance with the ethical standards of the responsible committee on human experimentation (institutional and national) and the Helsinki Declaration of 1975, as revised in 2008. Ethics committee approval was granted by our institution (protocol number 2019/1908, and informed consent was obtained from all participants. This trial was registered at ClinicalTrials.gov (NCT06283342).

### 2.6. Statistical analysis

Patient data collected within the scope of the study were analyzed using the IBM Statistical Package for the Social Sciences (SPSS) for Windows 26.0 (IBM Corp., Armonk) package program. Frequencies and percentages for categorical data and means and standard deviations for continuous data are presented as descriptive values. For comparisons between groups, the *“Independent Sample T-test”* was used for 2 groups, and the *“Pearson Chi-Square Test”* was used to compare categorical variables. Risk analysis was performed using Pearson’s chi-square test to determine the risk factors that play a role in ID and IDA as odds ratios (OR) and 95% confidence intervals. The results were considered statistically significant when the *P*-value was <0.05.

## 3. Results

Two hundred-four patients were included in this study. Of these patients, 91 (44.6%) were female and 113 (55.4%) were male. The mean age of the study group was 14.36 months, and the median age was 14 months. In our study, the ID rate was 53.9% (n = 110), and the IDA rate was 34.8% (n = 71). In the evaluation of the growth parameters of the patients according to the Centers for Disease Control and Prevention curve, the rate of low body weight for age was 22.1% (n = 45), and the rate of short stature for age was calculated as 4.9% (n = 10).

When the rates of microcytosis, transferrin saturation <15, and RDW values >16 in patients with and without IDA in the study group were compared statistically, the presence of microcytosis, transferrin saturation <15, and RDW values >16 were significantly higher in the IDA group (*P* = .001).

The rate of recommendation for iron prophylaxis in the 204 patients in our study was 95.1% (n = 194). The rate of prophylaxis recommended at the right time was 38.2% (n = 78) and the rate of being recommended at the correct dose was 42.2% (n = 86). In our study, the rate of prophylactic use among all patients was 66.7% (n = 136). However, the proportion of patients who correctly used prophylaxis in the entire group was 13.7% (n = 28). The breastfeeding rate for the 1st 6 months after delivery was 80.9% (n = 165).

Table 1 summarizes the evaluation and statistical analysis of the patient groups with ID and IDA in our study regarding prophylaxis use characteristics, breast milk intake, and adequate supplementary food intake in terms of iron. Accordingly, using prophylaxis correctly, consuming sufficient breast milk, and consuming supplementary food in terms of iron were statistically significant in preventing ID and IDA (*P* > .05).

**Table 1:** The relationship between iron prophylaxis, nutrition, and breast milk intake in relation to iron deficiency.

	ID	ID (not)	*P*-value	IDA	IDA (not)	*P*-value
Prophylaxis use	(53) 48.2%	(83) 88.3%	**.001**	(22) 31%	(114) 85.7%	**.001**
Prophylaxis not use	(57) 51.8%	(11) 11.7%		(49) 69%	(19) 14.3%	
Prophylaxis correctly	(6) 5.5%	(22) 23.4%	**.001**	(0) 0%	(28) 21.1%	**.001**
Prophylaxis not correctly	(104) 94.5%	(72) 76.6%		(71) 100%	(102) 78.9%	
Enough breastmilk	(83) 75.5%	(82) 87.2%	**.033**	(52) 73.2%	(113) 85%	**.043**
Insufficient breastmilk	(27) 24.5%	(12) 12.8%		(19) 26.8%	(20) 15%	
Iron-rich supplementary food	(31) 28.2%	(50) 53.2%	**.001**	(19) 26.8%	(62) 46.6%	**.006**
Iron-poor supplementary food	(79) %71.8	(44) 46.8%		(52) 73.2%	(71) 53.4%	

Bold values indicate significance at *P* < .05.

ID = iron deficiency, IDA = iron deficiency anemia.

The results of the risk analysis performed using Pearson’s Chi-square test at a 95% confidence interval to determine the effect of the risk factors of not using prophylaxis, insufficient breast milk, and insufficient supplementary food for iron on the development of ID and IDA are presented in Table 2. Accordingly, the most critical risk factor for developing ID and IDA was not prophylactic use (*P* = .001 and OR: 8.115 for ID and OR: 13.364 for IDA).

**Table 2:** The relationship between risk factors and iron deficiency and iron deficiency anemia.

	ID (110)			IDA (71)		
	%	*P*-value	OR	%	*P*-value	OR
Prophylaxis not use	51.8%	**.001**	8.115	69%	**.001**	13.364
Insufficient breast milk	24.5%	**.033**	5.296	26.8%	**.043**	2.064
Iron-poor supplementary food	71.8%	**.001**	2.889	73.2%	**.006**	2.390

Bold values indicate significance at *P* < .05.

ID = iron deficiency, IDA = iron deficiency anemia, OR = odd ratio.

The evaluation of the relationship between growth, ID, and IDA is presented in Table 3. A statistically significant relationship was observed between low body weight, ID, and IDA (*P* < .05). However, there was no statistically significant correlation between short stature, ID, and IDA (*P* > .05).

**Table 3:** The relationship between iron deficiency. iron deficiency anemia. and the growth of patients in terms of height and weight.

	ID (110)	ID not (94)	*P*-value	IDA (71)	IDA not (133)	*P*-value
Low body weight	(37) 33.6%	(73) 66.4%	**.001**	(25) 35.2%	(20) 15%	**.001**
Normal Body Weight	(8) 8.5%	(86) 91.5%		(46) 64.8%	(113) 85%	
Short stature	(7) 6.4%	(3) 3.2%	**.296**	(6) 8.5%	(4) 3%	**.086**
Reguler Stature	(103) 93.6%	(91) 96.8%		(65) 91.5%	(129) 97%	

Bold values indicate significance at *P* < .05.

ID = iron deficiency, IDA = iron deficiency anemia.

## 4. Discussion

The incidence of IDA during childhood exhibits 2 peaks. The 1st is at 6 to 24 months of age, and the other is adolescence.^[2]^ As a government policy in our country, 10 mg/day of prophylactic iron drops is recommended for children aged 4 to 12 months (*Iron-Like Turkey Program*), and the preparation is distributed free of charge. However, no such application is available for adolescents. Therefore, children between the ages of 6 and 24 months were included in this study. According to World Health Organization data, half of the world’s child population is affected by anemia and anemia-related diseases. In this context, our country is in the *“medium-risk countries”* group. The World Health Organization and the American Academy of Pediatrics recommend that infants be given iron supplements at 4 months of age in developing countries, where IDA is common.^[11,12]^ The median age of the patients in our study was 14 months. Most patients included in the study completed the prophylaxis period. Therefore, our study was valuable in showing the effects of prophylaxis on ID and IDA in the 6 to 24-month age range.

In an epidemiological study conducted by Gür et al,^[8]^ the prevalence of IDA in preschool children in Turkey was found to be 6% to 40% due to regional differences. In an epidemiological study covering the whole of Turkey, the frequency of ID was found to be 28.7%, and the frequency of IDA was 6.3% in children aged 6–17 months.^[9]^ The prevalence of anemia was 7.8% in 12- to 23-month-old children for whom the *Iron-Like Turkey Program* was effectively implemented.^[12]^ In our study, ID and IDA rates were 53.9% and 34.8%, respectively. In our study, the rates of ID and IDA were higher than those reported previously. We believe that the most important reason for the high rates of ID and IDA in our study is that the study population consisted of patients who applied to the hospital. We believe that the most important deficiency of our study is the absence of healthy and randomly selected children in the study.

IDA is a type of microcytic anemia. In our study, an MCV value of <70 µm^3^ (*microcytosis*), an RDW value of > 16 (*anisocytosis*), and transferrin saturation of <16 were significantly more common in patients with IDA than in those without IDA (*P* < .05). In our study, the MCV, RDW, and transferrin saturation values in the IDA group were consistent with those reported previously. They interpreted this as the correct diagnosis of our patients who were accepted as having IDA.

When the use of iron prophylaxis is examined in the literature, the rate of prophylaxis is in the range of 78.5% to 95%.^[10,13]^ Our literature review revealed no data on the rate of recommended prophylaxis. In our study, the rate of recommending iron prophylaxis was 95.1%, and the rate of prophylaxis use was 66.7%. The rate of prophylactic use was lower than that reported in literature. Iron prophylaxis reduces the rate of IDA.^[11]^ A meta-analysis investigating the benefit and safety of iron prophylaxis in children aged 4 to 24 months stated that iron supplementation reduced the development of IDA in this age group.^[7]^ In our study, the rates of not using prophylaxis in the ID and IDA patient groups were 51.8% and 69%, respectively. These rates were significantly lower than those in patients without ID or IDA (*P* < .05). Although the rate of prophylaxis use is mentioned in the literature, there are no data on the correct use of prophylaxis.^[10]^ In our study, the rate of correct prophylactic use was 13.7%. IDA was not detected in any of the patients who correctly used prophylaxis.

Breastfeeding in the 1st 6 months of life was found to be protective against ID and IDA.^[14]^ In our study, consistent with the literature, ID and IDA rates were found to be significantly higher in patients who did not receive breast milk in the 1st 6 months compared to those who did (*P* < .05). Protein-rich foods, such as beef, mutton, and chicken, are rich in heme iron. In addition to meat, well-cooked legumes, soybeans, eggs, dried fruits (*especially raisins and dried apricots*), molasses, green vegetables (*spinach*), nuts, peanuts, sesame, and tahini are rich in iron.^[15,16]^ The present study investigated the intake of red meat, eggs, dried legumes, and other iron-rich foods. ID and IDA were significantly lower in children who received adequate supplementary iron-rich food than in those who did not (*P* < .05).

In a meta-analysis conducted on children with IDA, dietary interventions were significant in treating IDA because of the comparison of iron therapy alone with iron therapy and dietary changes.^[17]^ However, it is unclear which method is better for IDA prevention. In recent years, the opinion that ID can be prevented through nutrition has not been clearly stated in the literature. The General Directorate of Maternal and Child Health and Family Planning of the Ministry of Health has provided free iron supplementation to babies aged 4 to 12 months for 5 months since 2004, aiming to reduce the frequency of IDA. In a field study conducted in 2009 to see the results of iron supplementation application, it was observed that iron supplementation was recommended to three-quarters of the children, and almost all families followed the recommendations. While two-thirds of the children included in the study who had their blood counts done before the study were diagnosed with anemia, as a result of this study, only 7.8% of the children aged 12 to 23 months were found to be anemic. Prophylactic iron treatment is still being applied to children aged 6 to 12 months under the *“Iron-Like Turkey”* program.^[18]^ However, in another study conducted in Istanbul, the frequency of IDA was 45% in the 9th month in children who started iron prophylaxis at the 4th month and 33% in the 24th month in children whose iron supplementation was stopped at the 12th month.^[19]^

In our study, lack of prophylaxis, breast milk for the 1st 6 months, and insufficient intake of iron-rich supplementary foods were associated with the development of ID and IDA. Risk analysis of these factors regarding the development of ID and IDA revealed that the most critical risk factor was not prophylactic use (*P* < .05, OR: 13.364). Insufficient intake of iron-rich supplementary food and not taking breast milk for the first 6 months were also risk factors for IDA and IDA (*P* < .05, OR: 2.390–2.06).

## 5. Conclusion

Prophylaxis emerged as the most critical risk factor for ID and IDA in children aged 6 to 24 months. Despite the solid recommendations, the compliance rate remains low. However, consuming more iron-rich foods and breastfeeding for the 1st 6 months is a reliable protective measure against ID and IDA, providing reassurance and confidence in preventing these conditions.

## Author contributions

**Conceptualization:** Ayşe Şimşek, Vesile Meltem Energin.

**Data curation:** Ayşe Şimşek.

**Formal analysis:** Ayşe Şimşek.

**Funding acquisition:** Ayşe Şimşek.

**Investigation:** Ayşe Şimşek.

**Methodology:** Ayşe Şimşek, Vesile Meltem Energin.

**Project administration:** Ayşe Şimşek.

**Resources:** Ayşe Şimşek.

**Software:** Ayşe Şimşek.

**Supervision:** Vesile Meltem Energin.

**Validation:** Ayşe Şimşek.

**Visualization:** Ayşe Şimşek.

**Writing – original draft:** Ayşe Şimşek, Vesile Meltem Energin.

**Writing – review & editing:** Ayşe Şimşek, Vesile Meltem Energin.
